# Impact of Germline Depletion of Bonus on Chromatin State in *Drosophila* Ovaries

**DOI:** 10.3390/cells12222629

**Published:** 2023-11-15

**Authors:** Baira Godneeva, Katalin Fejes Tóth, Baiyi Quan, Tsui-Fen Chou, Alexei A. Aravin

**Affiliations:** 1 Division of Biology and Biological Engineering, California Institute of Technology, Pasadena, CA 91125, USA; 2 Institute of Gene Biology, Russian Academy of Sciences, Moscow 119334, Russia; 3 Proteome Exploration Laboratory, Beckman Institute, California Institute of Technology, Pasadena, CA 91125, USA

**Keywords:** H3K9ac, H3K27ac, H3K27me3, transcription, Bonus, gene expression, chromatin

## Abstract

Gene expression is controlled via complex regulatory mechanisms involving transcription factors, chromatin modifications, and chromatin regulatory factors. Histone modifications, such as H3K27me3, H3K9ac, and H3K27ac, play an important role in controlling chromatin accessibility and transcriptional output. In vertebrates, the Transcriptional Intermediary Factor 1 (TIF1) family of proteins play essential roles in transcription, cell differentiation, DNA repair, and mitosis. Our study focused on Bonus, the sole member of the TIF1 family in *Drosophila*, to investigate its role in organizing epigenetic modifications. Our findings demonstrated that depleting Bonus in ovaries leads to a mild reduction in the H3K27me3 level over transposon regions and alters the distribution of active H3K9ac marks on specific protein-coding genes. Additionally, through mass spectrometry analysis, we identified novel interacting partners of Bonus in ovaries, such as PolQ, providing a comprehensive understanding of the associated molecular pathways. Furthermore, our research revealed Bonus’s interactions with the Polycomb Repressive Complex 2 and its co-purification with select histone acetyltransferases, shedding light on the underlying mechanisms behind these changes in chromatin modifications.

## 1. Introduction

Gene expression is a fundamental process that requires complex regulation and exhibits significant differences across tissues and cell types. As cells develop into specific types, they make important decisions that ultimately determine their unique cellular fates. Such a cell-specific form of gene expression is regulated via the presence of specific transcription factors, the dynamic landscape of chromatin modifications, and the activity of chromatin-regulating factors. To achieve precise regulation, these regulatory factors often work together. Histone marks are important regulators that determine chromatin accessibility and influence transcriptional outcomes [1,2,3,4]. These modifications act as a molecular code that is deciphered via specialized ‘reader’ proteins and associated complexes to determine the transcriptional output and functional characteristics of genomic regions. Histone acetylation, particularly H3K9ac and H3K27ac, is commonly associated with gene activation and the establishment of an open chromatin structure conducive to transcription [5,6,7]. In contrast, H3K27me3 is a hallmark of gene silencing and is closely associated with facultative heterochromatin and transcriptional repression [8,9,10,11]. H3K27me3 is deposited via the Polycomb Repressive Complex 2 (PRC2) and distributed across extensive genomic regions, referred to as Polycomb domains, to suppress cell type–specific expression programs [12,13,14,15]. The regulatory mechanisms that determine the precise targeting of histone-modifying enzymes to genomic sites remain poorly understood. Transcriptional Intermediary Factor 1 (TIF1) family proteins, known as chromatin-associated factors, have the ability to both activate and repress transcription by interacting with co-regulators and controlling the chromatin state [16,17,18,19,20]. Bonus (Bon) is the sole member of the TIF1 family in *Drosophila* [21]. Bon encompasses all the conserved domains present in mammalian members of the TIF1 family, including an N-terminal RBCC motif (composed of a RING domain, followed by two B-boxes, and a coiled coil domain) and a C-terminal chromatin-binding unit comprising a PHD domain and a bromodomain [17,21,22,23]. Previous investigations have highlighted the important role of Bon in embryonic development and organogenesis [21,24,25,26,27]. Our recent work unraveled Bon’s role as a regulator of tissue-specific genes within the female germline, revealing the importance of its SUMO modification in transcriptional repression [28]. In this study, we explored the role of Bon in directing different activating and silencing epigenetic modifications. Specifically, we examined the role of Bon in the distribution of the H3K27me3, H3K9ac, and H3K27ac marks. We demonstrated that the depletion of Bon in the ovaries leads to changes in the H3K27me3 landscape over transposon regions and an altered distribution of H3K9ac marks across specific protein-coding genes. Furthermore, we identified novel interacting partners of Bon, such as the PRC2 complex, PolQ, chromatin-binding proteins, and histone acetyltransferases, providing mechanistic insight on how these changes in chromatin modifications are achieved. Together, this study contributes to a deeper understanding of Bon’s involvement in epigenetic regulatory mechanisms.

## 2. Materials and Methods

### 2.1. Drosophila Fly Stocks

All fly stocks and crosses were raised at 24 °C. Females were 0–1 days old and were dissected right away. To obtain the fly lines with Bon knockdown, the short hairpin sequences were ligated into the pValium20 vector, and then integrated into the attP2 landing site (BDSC #8622). The UASp-λN-GFP fly line control was previously described [29]. To generate the UASp-λN-GFP-Bonus fly line, full-length cDNA sequences of wild-type Bon was cloned in vectors containing a miniwhite marker followed by the UASp promoter sequence and λN-GFP. Transgenic flies carrying these constructs were generated via phiC31 transformation by BestGene Inc and were integrated into the attP40 landing site (y1 w67c23; P{CaryP}attP40). The expression of these constructs was driven via the maternal alpha-tubulin67C-Gal4 (*MT-Gal4*) (BDSC #7063) and *nos-Gal4* (BDSC #4937) drivers.

### 2.2. S2 Cell Line

*Drosophila* S2 cells (DGRC catalog #006) were cultured at 25 °C in Schneider’s *Drosophila* medium supplemented with 10% heat-inactivated FBS and 1X penicillin–streptomycin.

### 2.3. Protein Co-Immunoprecipitation from S2 Cells

S2 cells were transfected with plasmids encoding GFP- and FLAG-tagged proteins under the control of the actin promoter using TransIT-LT1 reagent (Mirus, Madison, WI, USA). Around 35–40 h after transfection, cells were collected and resuspended in lysis buffer (composed of 20 mM of Tris-HCl (pH 7.4), 150 mM of NaCl, 0.2% NP-40, 0.2% Triton-X100, 5% glycerol, 20 mM of N-Ethylmaleimide (NEM) (Sigma, St. Louis, MO, USA), and complete protease inhibitor cocktail (Roche, Basel, Switzerland)). The cell lysate was incubated on ice for 20 min, centrifuged, and the supernatant was subsequently collected. The supernatant was incubated with magnetic agarose GFP-Trap beads (Chromotek, Planegg, Germany) for 3 h at 4 °C with end-to-end rotation. The beads were washed four times for 10 min with wash buffer (20 mM of Tris-HCl (pH 7.4), 0.1% NP40, and 150 mM of NaCl) and boiled in 2× Laemmli buffer for 5 min at 95 °C. The eluate was used for Western blot analysis.

### 2.4. Western Blotting

Proteins were separated via SDS-PAGE gel electrophoresis and transferred to a 0.45-μm nitrocellulose membrane (Bio-Rad, Hercules, CA, USA), according to standard procedures. The membrane was blocked with 0.2% I-block (Invitrogen, Carlsbad, CA, USA) in PBST (PBS, 0.1% Tween-20) for 1 h. The membrane was incubated with primary antibodies overnight at 4 °C, followed by 3× washes for 5 min in PBST, and incubation with secondary antibodies for 2 h at room temperature. The membrane was washed three times for 5 min with PBST and then imaged with the Odyssey system (Li-Cor, Lincoln, NE, USA). When the primary antibodies were HRP-conjugated, the membrane was washed 3 × 5 min with PBST and incubated with the HRP substrate; an X-ray film developed on an X-ray Film Processor (Konica Minolta, Tokyo, Japan). The following antibodies were used: HRP-conjugated anti-FLAG (Sigma, A8592, 1 mg/mL, dilution: 1:10,000), mouse anti-FLAG (Sigma, F1804, 1 mg/mL, dilution: 1:5000), rabbit polyclonal anti-GFP (dilution: 1:4000) [29], and IRDye anti-rabbit and anti-mouse secondary antibodies (Li-Cor, #925-68070 and #925-32211, 1 mg/mL, dilution: 1:10,000).

### 2.5. RNA Extraction and RNA-Seq Analysis

For RNA extraction, 10–20 pairs of dissected ovaries from lines with *BonusKD* driven by *nos-Gal4* (and matched siblings that lack the shRNA as control) were homogenized in TRIzol (Invitrogen); RNA was extracted, isopropanol precipitated, and treated with DNaseI (Invitrogen), according to the manufacturer’s instructions.

For RT-qPCR, reverse transcription was performed using random hexamer oligonucleotides with Superscript III Reverse Transcriptase (Invitrogen). qPCR was performed on a Mastercycler^®^ep realplex PCR machine (Eppendorf, Hamburg, Germany). Three biological replicates per genotype were used for all RT-qPCR experiments. Bon expression was normalized to rp49 mRNA expression. The data were visualized using Python 3 via JupyterLab. The following primers were used for qPCR analysis: bon-for: ACTTCTGGGTCTGACTGGCGAAG; bon-rev: TCAACGCACCACGACGTGG; rp49-for: CCGCTTCAAGGGACAGTATCTG; and rp49-rev: ATCTCGCCGCAGTAAACGC.

For RNA-seq libraries, PolyA+ selection was performed using a NEBNext Poly(A) mRNA Magnetic Isolation Module (NEB, #E7490). RNA-seq libraries were made using the NEBNext Ultra II Directional RNA Library Prep kit for Illumina (NEB, #E7760), according to the manufacturer’s instructions. Libraries were sequenced on the Illumina HiSeq 2500 platform. For RNA-seq coverage tracks, reads were first aligned to the *D. melanogaster* genome (dm6) using bowtie1 (v.1.2.2) allowing 2 mismatches and single mapping positions. Tracks were generated using the deepTools (v.3.5.1) bamCoverage function with 10 bp bin sizes.

### 2.6. ChIP-Seq

ChIP experiments were performed in two biological replicas for the H3K27ac and H3K9ac marks and in one replica for the H3K27me3 and H3K9me3 marks, as previously described [30]. In brief, 80–120 pairs of dissected ovaries were crosslinked with 1% formaldehyde in PBS for 10 min at room temperature, and then quenched with glycine (final concentration 25 mM). Frozen ovaries were dounced in RIPA buffer and then sonicated (Bioruptor sonicator) to a desired fragment size of 200–800 bp. Lysates were centrifuged at 19,000× *g*, and supernatants were collected. The supernatants were first precleared for 2 h at 4 °C using Protein G Dynabeads (Invitrogen). Precleared samples were immunoprecipitated with anti-H3K27me3 (C36B11, Cell Signaling), anti-H3K9me3 (ab8898, abcam), anti-H3K27ac (ab4729, abcam), or anti-H3K9ac antibodies (ab10812, abcam) for 3–5 h at 4 °C; then, 50 μL of Protein G Dynabeads were added, and the samples were further incubated overnight at 4 °C. The beads were washed 3× 10 min in LiCL buffer, followed by proteinase K treatment for 2 h at 55 °C and then overnight at 65 °C. DNA was extracted via standard phenol/chloroform extraction. ChIP-seq libraries were prepared using NEBNext Ultra DNA Library Prep Kit Illumina and sequenced on the Illumina HiSeq 2500 platform (SR 50 bp for H3K27me3 and H3K9me3; PE 50bp for the H3K27ac mark) and NovaSeq 6000 platform (PE150 for the H3K9ac mark). After removal of the adaptors, reads with a minimal length of 18 nucleotides were aligned to the *D. melanogaster* genome (dm6) using bowtie1 (v.1.2.2) allowing 2 mismatches and single mapping positions. Protein-coding gene annotations and their repetitive sequence annotations were obtained from the RefSeq and RepeatMasker tables, respectively, retrieved from the UCSC genome browser. Genome coverage tracks were generated using the deepTools (v.3.5.1) bamCoverage function with 10 bp bin sizes. The ChIP signal was normalized to input counts by calculating the cpm (counts per million) using the deepTools bamCompare function with 50 bp bin sizes (log2 values ChIP/Input). Heatmaps and plot profiles were generated with deepTools plotHeatmap and plotProfile using log2-normalized (ChIP/Input) BigWig files.

### 2.7. Liquid Chromatography–Mass Spectrometry (LS–MS)

All procedures were performed using MS-grade water, low binding plasticware, and freshly prepared solutions. Dissected ovaries from 1–2-day old *Drosophila* flies expressing UASp-λN-GFP-Bonus or UASp-λN-GFP (control) were used for MS. Frozen ovaries were dounced in lysis buffer (20 mM of Tris-HCl (pH 7.4), 150 mM of NaCl, 10% glycerol, 0.5% DMM (n-Dodecyl-β-D-maltoside), and 25 mM of NEM (N-Ethylmaleimide), supplied with protease inhibitor). Lysates were centrifuged at 19,000× *g*, and the supernatants were collected. The supernatants were then precleared for 2 h at 4 °C using Protein G Dynabeads (Invitrogen). Precleared samples were immunoprecipitated with anti-GFP antibody for 4 h at 4 °C; then, 50 μL of Protein G Dynabeads were added, and the samples were further incubated overnight at 4 °C. The beads were washed 5× 10 min in lysis buffer, and then 2× 5 min in wash buffer (50 mM HEPES, pH 8.0). Then, samples were eluted from the beads with 10 M urea for 15 min at 37 °C. After that, the samples were diluted to 8M urea with wash buffer. After that, the eluates were incubated with 500 mM of TCEP (Thermo Scientific, Waltham, MA, USA, 20490) for 20 min at 37 °C, and then with 500 mM of 2-chloroacetamide for 15 min at 37 °C; after that, the samples were incubated with Endoproteinase LysC for 4 h at 37 °C. The samples were diluted to final 2 M urea with wash buffer; after that, 100 mM of CaCl2 with trypsin (100 ng/μL) was added for overnight incubation at 37 °C. For desalting, C18 spin columns (Thermo Scientific, 89870) were used, according to the manufacturer’s instructions. After elution, the samples were freeze dried and submitted to the Caltech Proteome Exploration Laboratory for MS analysis. Peptide samples were subjected to LC-MS analysis on an EASY-nLC 1200 (Thermo Fisher Scientific) coupled to a Q Exactive HF Orbitrap mass spectrometer. Raw data files were searched against a customized database (*Drosophila melanogaster* and bait proteins) using the Proteome Discoverer 2.5 software based on the SEQUEST algorithm. The fragment mass tolerance was set to 20 ppm. The maximum false peptide discovery rate was specified as 0.01 using the Percolator Node validated by a *q*-value.

Gene ontology (GO) molecular function term enrichment analysis was performed on proteins that were significantly enriched compared to the control (log2FC > 1, adjusted *p*-value < 0.05, Benjamini–Hochberg), using DAVID Bioinformatics Resources. The enriched GO terms associated with 2 or less submitted genes were excluded. A significant threshold was applied using a multiple testing correction (Fisher’s Exact test *p*-value < 0.01). Data visualization was performed using standard plotting libraries using Python (version 3.9.15).

## 3. Results

### 3.1. Genome-Wide Analyses of H3K27me3 Distribution in Bonus-Depleted Ovaries

**Figure 1:**
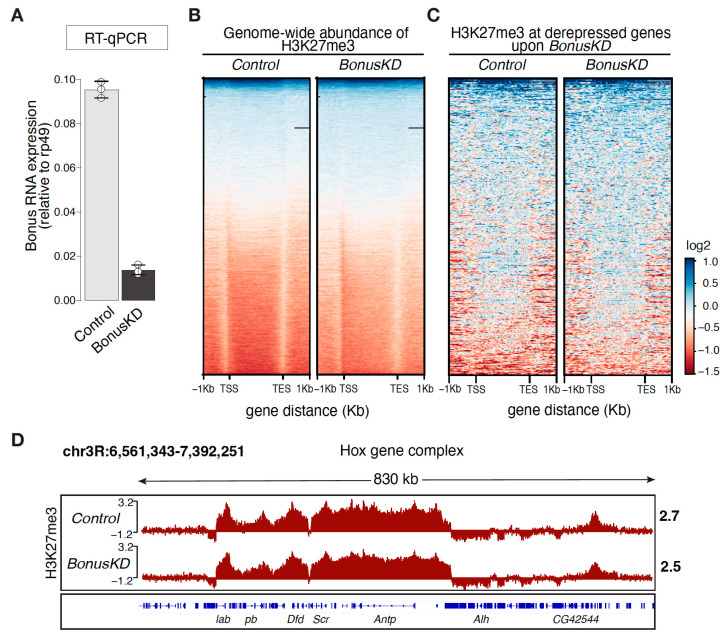
Distribution of the H3K27me3 mark in Bon-depleted ovaries. (**A**) Bar graph shows the relative expression of Bon (normalized to rp49 level) in control and Bon-depleted ovaries (RT-qPCR; dots correspond to 3 independent biological replicates; error bars indicate the st. dev.). (**B**) Genome-wide abundance of H3K27me3 over the gene body in control and Bon germline knockdown ovaries (*BonusKD*). The heatmap displays the distribution of H3K27me3 ± 1 kb over the gene body in control and *BonusKD* ovaries (input-normalized log2 values). (**C**) Heatmap showing H3K27me3 distribution across Bon targets in control and *BonusKD* ovaries (input-normalized log2 values). (**D**) Distribution of H3K27me3 over the Hox gene complex Antp. Tracks show the CPM-normalized coverage of ChIP-seq data for the H3K27me3 mark in control and *BonusKD* ovaries (log2 values). Numbers show the normalized log2 values of the ChIP/input signal (ChIP-seq) in a manually selected genomic location. The bottom panel shows the structure of the gene; blue arrows indicate the direction of transcription.

To gain insight into Bon’s role within the epigenetic landscape, we first performed ChIP-seq analysis of genome-wide distribution of repressive H3K27me3 marks in Bon-depleted ovaries. We used ovaries from flies expressing short hairpin RNAs against *bon* using a *nos-Gal4* driver to achieve the germline-specific knockdown of Bon (*BonusKD*). As a control, we used ovaries from their siblings that lack the short hairpin. This approach, utilizing siblings as controls, was chosen to minimize genetic variability. In a previous study, we demonstrated the effectiveness of our knockdown system, showing an 88% reduction in Bon gene expression via RT-qPCR and confirming this reduction in germ cells using confocal imaging [28]. In our current analysis, we verified the efficiency of Bon knockdown via RT-qPCR, revealing an 85% reduction in ovarian Bon expression, consistent with our previous findings (Figure 1A). The analysis of normalized H3K27me3 ChIP-seq signals across ± 1 kb of the transcription start sites (TSSs) and the transcription end site (TES) of all genes in the *Drosophila* genome revealed distinct genic distributions, with H3K27me3 covering the gene body in both the control and Bon-depleted ovaries (Figure 1B and Figure S1A). Bon depletion does not alter the levels of the H3K27me3 mark over protein-coding genes, including H3K27me3-decorated genes that become upregulated upon Bon knockdown, as demonstrated by RNA-seq (Figure 1C). To validate our ChIP-seq analysis, we examined the Hox gene clusters, well known for their spatial compartmentalization and significant enrichment of the H3K27me3 mark [31,32]. The Antennapedia (Antp) Hox gene complex, located on chromosome 3R in *Drosophila melanogaster*, displayed an unaltered and complete coverage of H3K27me3, confirming the validity of our ChIP-seq analysis and the lack of an effect following the germline-specific knockdown of Bon (Figure 1D). Therefore, our results suggest that repression of Bon-regulated genes is independent of the H3K27me3 mark.

### 3.2. Depletion of Bonus in the Ovaries Affects the Distribution of the H3K27me3 Mark over Transposon Regions

**Figure 2:**
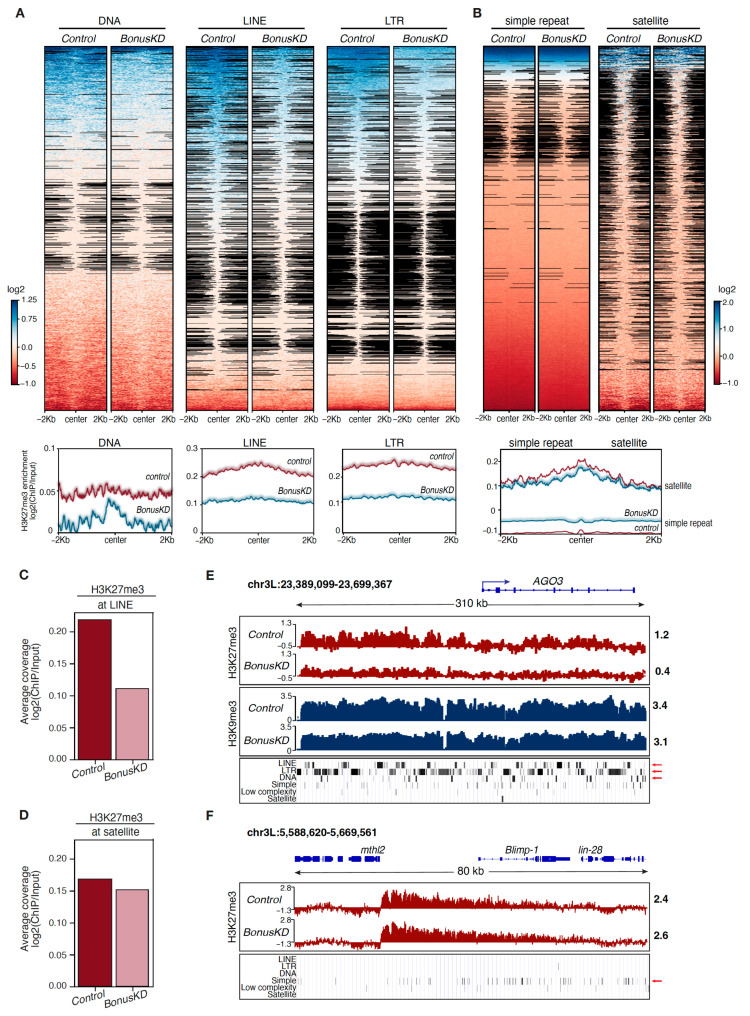
Differential impact of Bonus depletion on H3K27me3 distribution over transposon regions. (**A**) Heatmaps show H3K27me3 distribution at DNA repeat elements (DNA), long interspersed nuclear elements (LINE), and long terminal repeat elements (LTR) determined via RepeatMasker in control and *BonusKD* ovaries. Bottom panels show the average profiles of H3K27me3 in control and *BonusKD* ovaries over indicated regions. (**B**) Heatmaps show H3K27me3 distribution at simple repeats (micro-satellites) and satellite repeats determined via RepeatMasker in control and *BonusKD* ovaries. Bottom panel shows the average profiles of H3K27me3 in control and *BonusKD* ovaries over indicated regions. (**C**) Bar plot shows the average H3K27me3 enrichment at LINE sequences in control and *BonusKD* ovaries (averaged normalized log2 values of the ChIP/input signal). (**D**) Bar plot shows the average H3K27me3 enrichment at satellite sequences in control and *BonusKD* ovaries (averaged normalized log2 values of the ChIP/input signal). (**E**) Example of a slight reduction in the H3K27me3 mark over a transposon region located upstream of the gene AGO3. Tracks show counts per million (CPM)-normalized coverage of ChIP-seq data for H3K27me3 and H3K9me3 marks in control and *BonusKD* ovaries (log2 values). The top panel shows the gene structure of AGO3; the blue arrow indicates the direction of transcription. Numbers show the normalized log2 values of the ChIP/input signal (ChIP-seq) in a manually selected genomic location. The bottom panel shows the RepeatMasker track; the red arrows highlight the LTR, LINE, and DNA repeats. (**F**) Example of H3K27me3 mark distribution over a region with simple repeats. Tracks show CPM-normalized coverage of ChIP-seq data for the H3K27me3 mark in control and *BonusKD* ovaries (log2 values). The top panel shows the gene’s structure; the blue arrows indicate the direction of transcription. The numbers indicate the normalized log2 values of the ChIP/input signal (ChIP-seq) in a manually selected genomic location. The bottom panel displays the RepeatMasker track; the red arrow highlights simple repeats.

The PRC2 complex has been widely recognized for its role in depositing the H3K27me3 histone mark on protein-coding genes, while transposable elements (TEs) are repressed via DNA methylation and/or the H3K9me3 mark. However, recent studies have revealed that H3K27me3 is enriched over TE sequences across distantly related eukaryotes [33,34,35,36]. We consequently expanded our analysis to explore the impact of Bon knockdown on the H3K27me3 profile of repetitive sequences. A closer examination of diverse repetitive sequences unveiled that Bon depletion led to a reduction in H3K27me3 occupancy over LTR and LINE retrotransposon sequences, as well as DNA transposons (Figure 2A). In contrast, highly repeated satellites and simple repeats revealed no significant alterations (Figure 2B). Additionally, when we compared the average H3K27me3 enrichment across all LINE sequences in Bon-depleted ovaries and the control ovaries, we noted a reduction in the H3K27me3 signal following Bon knockdown, unlike at satellites, where we did not observe any significant changes (Figure 2C,D). For example, the region upstream of the gene AGO3 that contains multiple LINE, LTR, and DNA transposons exhibited a significant depletion of the H3K27me3 mark following Bon depletion (Figure 2E). It is noteworthy that this region is also covered by the H3K9me3 repression mark, which remained largely unchanged following Bon depletion. Thus, though Bon influences the accumulation of H3K27me3 marks over transposon regions, the concurrent presence of H3K9me3 appears to be sufficient to repress their transcription (Figure 2E). In comparison, the accumulation of H3K27me3 at a distinct region enriched in simple repeats remained unaltered following Bon knockdown (Figure 2F). Taken together, our results indicate that Bon is required for the accumulation of the H3K27me3 mark across different classes of transposable elements, but not simple repeats.

### 3.3. Modulation of the H3K27ac and H3K9ac Marks in Response to Bon Depletion

**Figure 3:**
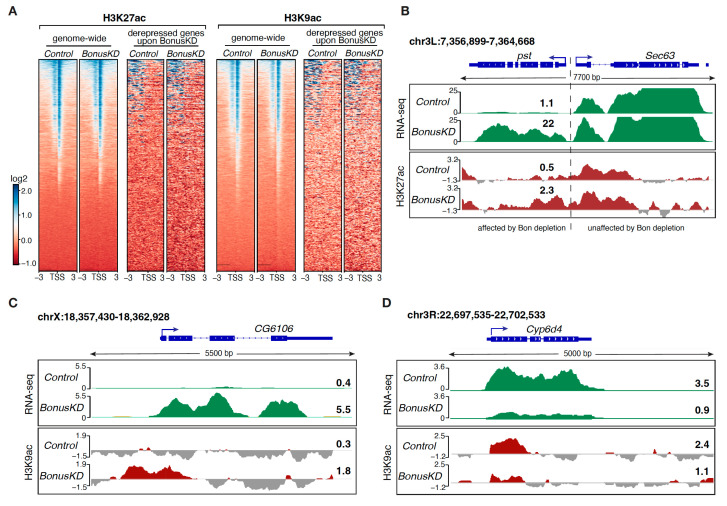
Enriched levels of the H3K27ac and H3K9ac marks on several Bon-regulated genes. (**A**) Distribution of H3K27ac and H3K9ac around the TSS in control and Bon germline knockdown ovaries (*BonusKD*). The right panel for each mark shows heatmaps indicating the distribution of H3K27ac and H3K9ac ± 3 kb around the TSS in control and *BonusKD* ovaries (input-normalized log2 values); the left panel shows heatmaps indicating the distribution of H3K27ac and H3K9ac across Bon targets in control and *BonusKD* ovaries (input-normalized log2 values). (**B**) RNA-seq and ChIP-seq tracks show CPM-normalized coverage for *pst* in control and *BonusKD* ovaries. The gene structure is depicted at the top; the arrow indicates the direction of transcription. The numbers indicate the CPM values of the exonic regions (RNA-seq) or the log2-normalized ChIP/input signal (ChIP-seq) in a manually selected genomic location. For ChIP-seq track, negative values are in a gray color. (**C**) The RNA-seq and ChIP-seq tracks show CPM-normalized coverage for *CG6106* in control and *BonusKD* ovaries. The gene structure is depicted at the top; the arrow indicates the direction of transcription. The numbers indicate the CPM values of the exonic regions (RNA-seq) or the log2-normalized ChIP/input signal (ChIP-seq) in a manually selected genomic location. For ChIP-seq track, negative values are in a gray color. (**D**) RNA-seq and ChIP-seq tracks show CPM-normalized coverage for *Cyp6d4* in control and *BonusKD* ovaries. The gene structure is depicted at the top; the arrow indicates the direction of transcription. The numbers indicate the CPM values of the exonic regions (RNA-seq) or the log2-normalized ChIP/input signal (ChIP-seq) in a manually selected genomic location. For ChIP-seq track, negative values are in a gray color.

After exploring the effect of Bon on repressive chromatin marks, we studied the acetylation of H3K27 and H3K9, which serve as catalysts for the establishment of open chromatin and are associated with active transcription. H3K27ac has been uncovered as a key player in cell identity control and a characteristic chromatin signature of active enhancers [37,38], while the H3K9ac mark is considered a hallmark of active gene promoters [39]. A genome-wide analysis revealed substantial occupancy of these activation marks at TSS and TSS-proximal regions, aligning with their known presence in the proximity of active promoters. Depleting Bon did not affect the overall distribution patterns of H3K27ac and H3K9ac of the majority of TSSs genome-wide (Figure 3A and Figure S1B,C); however, a subset of genes that become aberrantly expressed or silenced upon Bon depletion exhibited a concomitant accumulation or loss of these marks, respectively. For example, Bon depletion leads to ectopic activation of the *pst* gene and associated accumulation of the H3K27ac mark near TSS-proximal regions (Figure 3B), while activated *CG6106* displays H3K9ac accumulation at its TSS (Figure 3C). In contrast *Cyp6d4*, which experienced a stark decline in expression upon Bon depletion, exhibited a notable decrease in H3K9ac levels (Figure 3D). Overall, our results indicated that only a small fraction of Bon-regulated genes depend on the H3K9ac or H3K27ac activation marks. Interestingly, a gain or loss of the H3K9 and H3K27 acetylation signals at promoters did not correlate with the fold change in the corresponding genes’ expression level, pointing towards complex and context-dependent regulatory relationships that may involve multiple pathways and mechanisms.

### 3.4. Bonus Associates with Histone Acetyltransferases and the PRC2 Complex

**Figure 4:**
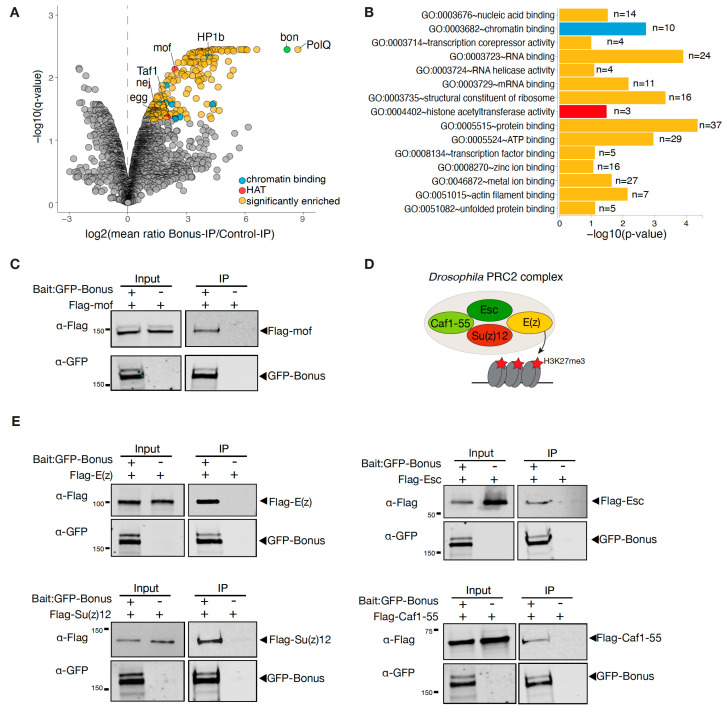
Bonus interacts with histone acetyltransferases and with the PRC2 complex. (**A**) Bon co-purifies with several proteins in the ovaries. Volcano plot showing fold enrichments versus statistical significance (determined via quantitative MS) of proteins in GFP-Bonus co-immunoprecipitants versus control (log2FC > 1, adjusted *p*-value < 0.05, Benjamini–Hochberg; 2 biological replicates; experimental flies express GFP-tagged Bon; flies expressing GFP served as a control). Bon and selected interacting proteins are labeled. (**B**) Gene ontology (GO) molecular function analysis of identified partners of Bon. Bar plot illustrates the analysis for proteins that were significantly enriched compared to the control (log2FC > 1, adjusted *p*-value <0.05, Benjamini–Hochberg). Only selected GO terms above the established cut-off criteria (*p*-value < 0.01 and >2 genes per group) are shown. The number of proteins associated with specific functional terms is displayed on the bars. (**C**) Bon interacts with MOF. Western blot analysis of immunoprecipitation experiment using GFP nanotrap beads from S2 cells co-expressing GFP-Bonus and Flag-tagged MOF. Lysate not expressing GFP-Bonus was used as a negative control. (**D**) Model showing subunits of the *Drosophila* PRC2 complex. (**E**) Bon interacts with components of the PRC2 complex: E(z), Su(z)12, Caf1-55, and Esc. Western blot analysis of immunoprecipitation experiment using GFP nanotrap beads from S2 cells co-expressing GFP-Bonus and Flag-tagged E(z), Flag-tagged Esc, Flag-tagged Su(z)12, and Flag-tagged Caf1-55. Lysates not expressing GFP-Bonus were used as negative controls.

In order to identify Bon’s interaction partners, we immunopurified GFP-tagged Bon from fly ovaries followed by quantitative mass spectrometry (MS). As a control, we used flies only expressing GFP. MS analyses were performed in two biological replicates. The MS analysis identified 315 proteins, with a false discovery rate lower than 5% after adjustments (Benjamini–Hochberg test). Among these, 230 candidate proteins were identified that were at least 2-fold more abundant in the GFP-Bon pulldown compared to the control, suggesting that they specifically associate with Bon (Figure 4A). We then employed gene ontology (GO) analysis using DAVID Bioinformatics Resources to predict the molecular functions of these identified partners. This detailed analysis revealed significant levels of enrichment chromatin-binding proteins, such as HP1b, Egg, and HDAC6 (Figure 4B). This observation firmly supports an important role for Bon in chromatin-related processes. It also aligns with our previously reported data, where we demonstrated the interaction of Bon with the H3K9 histone methyltransferase Egg/SetDB1 [28]. Interestingly, the strongest Bon interactor was PolQ, which is known for its role in a DNA double-strand break (DSB) repair mechanism [40,41,42]. Several identified proteins, such as Rox8, Srp54, and Spf30, harbor known RNA-binding activities and are documented to mostly participate in the regulation of mRNA splicing [43,44]. In addition, we found three distinct histone acetyltransferases (HATs) that were co-purifying with Bon (Figure 4A): (i) the histone acetyltransferase MOF, known to acetylate histone 4 at lysine 16 (H4K16ac) [45], (ii) Taf1, primarily engaged in acetylating histone 4 tails, and (iii) nej, responsible for the acetylation of H3K18 and H3K27 [46,47]. We further confirmed Bon’s interaction with MOF by co-expressing tagged MOF and Bon in S2 cells followed by immunoprecipitation (Figure 4C).

Additionally, we employed a different approach to explore a possible interaction between Bon and components of the PRC2 complex. In Drosophila, the PRC2 complex comprises four cardinal components: E(z), Su(z)12, Esc, and Caf1-55 (Figure 4D) [13,15,48,49]. We employed a co-immunoprecipitation assay using Flag-tagged proteins of the PRC2 complex and GFP-tagged Bon in S2 cells. Western blot analysis revealed that Bon co-purifies with E(z), Esc, Su(z)12, and Caf1-55 (Figure 4E). Based on these results, we concluded that Bon likely associates with the entire PRC2 complex. Overall, our results identified several novel binding partners for Bon.

## 4. Discussion

TIF1 family members play an important role in remodeling chromatin and the modulation of underlying transcriptional mechanisms [50]. Bon is the only member of the TIF1 subfamily of TRIM/RBCC proteins in *Drosophila*, setting it apart from its mammalian counterparts, where four distinct members exhibit diverse functions and mechanisms of action [16,17,18,19,20,21]. In this study, we performed a genome-wide analysis of several activating and silencing histone modifications in germline Bon-depleted ovaries. We found that Bon’s depletion did not significantly alter the genome-wide abundance of H3K27me3, although subtle reductions in H3K27me3 were observed in certain transposon-associated regions (Figure 1 and Figure 2). Our previous research indicated that Bon is not involved in transposon repression [28]. This can be explained that despite the mild reduction in H3K27me3, transposon expression remained largely unchanged due to the persistent presence of the repressive H3K9me3 mark (Figure 2E). While it has been well-established that H3K9me3 primarily marks transposable elements and satellite sequences [51,52], several studies have revealed the coexistence both the H3K9me3 and H3K27me3 marks on the same subset of genes [53,54], and in some the H3K27me3 mark is primarily responsible for the repression of transposons [33,34,35]. In mammals, TIF1β/KAP-1 is known to repress endogenous retroviruses in an H3K9me3-dependent way [18,19]. Although relatively few studies have showed the connection between the TIF1 family proteins and the H3K27me3 mark, one study did reveal that KAP-1 depletion resulted in a decreased level of H3K27me3 on the HIV-1 promoter [55]. Therefore, we propose that in *Drosophila*, Bon participates in the repression of transposons through its association with the H3K27me3 mark, indicating a new role within the TIF1 subfamily.

Additionally, the association between Bon and the PRC2 complex, revealed through co-immunoprecipitation assays, raises intriguing questions regarding the functional consequences of this interaction (Figure 4E). To the best of our knowledge, Bon has not previously been described in connection with Polycomb complexes, apart from a potential interaction between Bon and unknown members of a Polycomb group that was mentioned in the discussion of an early work [21]. Interestingly, in mammals, KAP-1 was shown to interact with EZH2/E(z) in a PRC2-independent manner to coordinately regulate genes with roles in breast stem cell maintenance [56]. In another study, KAP-1 associates with the PRC1 complex to repress differentiation-inducible genes in embryonic stem cells [57]. Therefore, Bon may be involved in facilitating the recruitment of the PRC2 complex to specific genomic loci or can also associate with the PRC1 complex to repress the subsets of several genes.

The members of the TIF1 family are involved in both transcriptional activation and repression. Our analysis of the active marks H3K9ac and H3K27ac indicates that Bon is directly regulating some of its targets by facilitating their H3K9 acetylation (Figure 3). However, the levels of these activation marks remained unchanged following Bon depletion on most of its targets, suggesting the involvement of other activation marks. Indeed, we did not identify the Gcn5 histone acetyltransferase responsible for the deposition of the H3K9ac mark in our mass-spec analysis for Bon interactors [58]. The presence of HATs MOF, nej, and Taf1 in the Bon-interacting complex raises the possibility that Bon’s modulation of gene expression may be accomplished through diverse acetylation marks (Figure 4A). Additionally, indirect regulation through other transcriptional regulators remains a plausible mechanism. Notably, due to their C-terminal PHD/bromodomain, TIF1 proteins can function as ‘readers’ of histone modifications. Thus, mammalian TIF1γ/TRIM33 specifically recognizes an H3 tail that is acetylated at lysines K18 and K23 [59], and TIF1α/TRIM24 was shown to recognize acetylated H3K23 [60]. Since our current results could not pinpoint a specific acetylation mark involved in Bon-dependent regulation, future studies will be necessary to precisely identify the acetylation mark associated with this process.

Our search for interacting partners of Bon revealed an interesting interaction with PolQ, a specialized DNA polymerase (Pol θ) participating in DNA repair, which suggests Bon’s potential involvement in end-joining repair of DSBs. In mammals, TIF1 members are known to play a major role in DSB repair mechanisms [61,62,63]. DSBs are serious threats to cell survival and genome stability; therefore, in the future, it will be interesting to determine whether Bon is required for the repair activities or whether it can enhance the efficiency and accuracy of this repair process, ensuring the preservation of genomic integrity.

In summary, our study expands our understanding of Bon’s roles in the epigenetic landscape and offers a valuable resource of Bon’s interactors in *Drosophila* ovaries. Although we demonstrate Bon’s influence on the distribution of activating marks (H3K9ac and H3K27ac) and a repressive mark (H3K27me3), it is essential to acknowledge the complexity of chromatin regulation. Therefore, it will be necessary to study other histone modifications and effector molecules to better understand the mechanisms underlying Bon’s impact on gene expression that controls the function of chromatin.

## 5. Conclusions

In conclusion, our study of the specific role of Bon, the only TIF1 family protein in *Drosophila*, has provided significant insights into its diverse contributions to the epigenetic landscape. Depletion of Bon affects the distribution of histone marks H3K27me3, H3K9ac, and H3K27ac, revealing its complex involvement in the regulation of chromatin states. The interaction with the PRC2 complex suggests potential involvement in Polycomb-mediated gene regulation, raising questions about the functional consequences of this cooperation. Our study reveals previously unknown interactions between Bon and chromatin-modifying complexes, including histone acetyltransferases. Furthermore, the dual role of Bon in transcriptional activation and repression suggests the existence of a complex regulatory network. While our study provides valuable insights into Bon’s interactions with key chromatin-modifying complexes and its role in DNA repair, the complex nature of chromatin regulation requires further exploration of additional histone modifications and effector molecules. Future studies, with a focus on identifying specific acetylation marks associated with Bon-dependent regulation and elucidating Bon’s role in DNA repair mechanisms, will enhance our understanding of its influence on gene expression and genome stability.

## Data Availability

The data presented in this study are available from the corresponding author on reasonable request.

## Supplementary Materials

The following supporting information can be downloaded at: https://www.mdpi.com/article/10.3390/cells12222629/s1, Figure S1: Average plot profiles of H3K27me3 (A), H3K27ac (B), and H3K9ac (C) over the gene body in control and Bon germline knockdown ovaries (*BonusKD*).

## References

[1] Turner B.M. (2005). Reading signals on the nucleosome with a new nomenclature for modified histones. Nat. Struct. Mol. Biol..

[2] Kouzarides T. (2007). Chromatin modifications and their function. Cell.

[3] Tan M., Luo H., Lee S., Jin F., Yang J.S., Montellier E., Buchou T., Cheng Z., Rousseaux S., Rajagopal N. (2011). Identification of 67 Histone Marks and Histone Lysine Crotonylation as a New Type of Histone Modification. Cell.

[4] Klemm S.L., Shipony Z., Greenleaf W.J. (2019). Chromatin accessibility and the regulatory epigenome. Nat. Rev. Genet..

[5] Grunstein M. (1997). Histone acetylation in chromatin structure and transcription. Nature.

[6] Choudhary C., Kumar C., Gnad F., Nielsen M.L., Rehman M., Walther T.C., Olsen J.V., Mann M. (2009). Lysine Acetylation Targets Protein Complexes and Co-Regulates Major Cellular Functions. Science.

[7] Bannister A.J., Kouzarides T. (2011). Regulation of chromatin by histone modifications. Cell Res..

[8] Margueron R., Justin N., Ohno K., Sharpe M.L., Son J., Iii W.J.D., Voigt P., Martin S.R., Taylor W.R., De Marco V. (2009). Role of the polycomb protein EED in the propagation of repressive histone marks. Nature.

[9] Yuan W., Wu T., Fu H., Dai C., Wu H., Liu N., Li X., Xu M., Zhang Z., Niu T. (2012). Dense Chromatin Activates Polycomb Repressive Complex 2 to Regulate H3 Lysine 27 Methylation. Science.

[10] Allis C.D., Jenuwein T. (2016). The molecular hallmarks of epigenetic control. Nat. Rev. Genet..

[11] Allshire R.C., Madhani H.D. (2018). Ten principles of heterochromatin formation and function. Nat. Rev. Mol. Cell Biol..

[12] Schuettengruber B., Chourrout D., Vervoort M., Leblanc B., Cavalli G. (2007). Genome Regulation by Polycomb and Trithorax Proteins. Cell.

[13] Schuettengruber B., Bourbon H.-M., Di Croce L., Cavalli G. (2017). Genome Regulation by Polycomb and Trithorax: 70 Years and Counting. Cell.

[14] Cao R., Wang L., Wang H., Xia L., Erdjument-Bromage H., Tempst P., Jones R.S., Zhang Y. (2002). Role of Histone H3 Lysine 27 Methylation in Polycomb-Group Silencing. Science.

[15] Müller J., Hart C.M., Francis N.J., Vargas M.L., Sengupta A., Wild B., Miller E.L., O’Connor M.B., Kingston R.E., Simon J.A. (2002). Histone methyltransferase activity of a Drosophila Polycomb group repressor complex. Cell.

[16] Nielsen A.L., Ortiz J.A., You J., Oulad-Abdelghani M., Khechumian R., Gansmuller A., Chambon P., Losson R. (1999). Interaction with members of the heterochromatin protein 1 (HP1) family and histone deacetylation are differentially involved in transcriptional silencing by members of the TIF1 family. EMBO J..

[17] Venturini L., You J., Stadler M., Galien R., Lallemand V., Koken M.H., Mattei M.G., Ganser A., Chambon P., Losson R. (1999). TIF1gamma, a novel member of the transcriptional intermediary factor 1 family. Oncogene.

[18] Schultz D.C., Friedman J.R., Rauscher F.J. (2001). Targeting histone deacetylase complexes via KRAB-zinc finger proteins: The PHD and bromodomains of KAP-1 form a cooperative unit that recruits a novel isoform of the Mi-2alpha subunit of NuRD. Genes Dev..

[19] Schultz D.C., Ayyanathan K., Negorev D., Maul G.G., Rauscher F.J. (2002). SETDB1: A novel KAP-1-associated histone H3, lysine 9-specific methyltransferase that contributes to HP1-mediated silencing of euchromatic genes by KRAB zinc-finger proteins. Genes Dev..

[20] Khetchoumian K., Teletin M., Mark M., Lerouge T., Cerviño M., Oulad-Abdelghani M., Chambon P., Losson R. (2004). TIF1δ, a Novel HP1-interacting Member of the Transcriptional Intermediary Factor 1 (TIF1) Family Expressed by Elongating Spermatids. J. Biol. Chem..

[21] Beckstead R., Ortiz J.A., Sanchez C., Prokopenko S.N., Chambon P., Losson R., Bellen H.J. (2001). Bonus, a Drosophila homolog of TIF1 proteins, interacts with nuclear receptors and can inhibit betaFTZ-F1-dependent transcription. Mol. Cell.

[22] Le Douarin B., Zechel C., Garnier J., Lutz Y., Tora L., Pierrat P., Heery D., Gronemeyer H., Chambon P., Losson R. (1995). The N-terminal part of TIF1, a putative mediator of the ligand-dependent activation function (AF-2) of nuclear receptors, is fused to B-raf in the oncogenic protein T18. EMBO J..

[23] Le Douarin B., Nielsen A.L., Garnier J.M., Ichinose H., Jeanmougin F., Losson R., Chambon P. (1996). A possible involvement of TIF1 alpha and TIF1 beta in the epigenetic control of transcription by nuclear receptors. EMBO J..

[24] Allton K., Jain A.K., Herz H.-M., Tsai W.-W., Jung S.Y., Qin J., Bergmann A., Johnson R.L., Barton M.C. (2009). Trim24 targets endogenous p53 for degradation. Proc. Natl. Acad. Sci. USA.

[25] Beckstead R.B., Ner S.S., Hales K.G., Grigliatti T.A., Baker B.S., Bellen H.J. (2005). Bonus, a Drosophila TIF1 homolog, is a chromatin-associated protein that acts as a modifier of position-effect variegation. Genetics.

[26] Ito H., Sato K., Koganezawa M., Ote M., Matsumoto K., Hama C., Yamamoto D. (2012). Fruitless Recruits Two Antagonistic Chromatin Factors to Establish Single-Neuron Sexual Dimorphism. Cell.

[27] Zhao H., Moberg K.H., Veraksa A. (2023). Hippo pathway and Bonus control developmental cell fate decisions in the Drosophila eye. Dev. Cell.

[28] Godneeva M.B., Ninova M., Tóth K.F., Aravin A.A. (2023). SUMOylation of Bonus, the Drosophila homolog of Transcription Intermediary Factor 1, safeguards germline identity by recruiting repressive chromatin complexes to silence tissue-specific genes. eLife.

[29] Ninova M., Chen Y.-C.A., Godneeva B., Rogers A.K., Luo Y., Tóth K.F., Aravin A.A. (2020). Su(var)2-10 and the SUMO Pathway Link piRNA-Guided Target Recognition to Chromatin Silencing. Mol. Cell.

[30] Le Thomas A., Stuwe E., Li S., Du J., Marinov G., Rozhkov N., Chen Y.-C.A., Luo Y., Sachidanandam R., Toth K.F. (2014). Transgenerationally inherited piRNAs trigger piRNA biogenesis by changing the chromatin of piRNA clusters and inducing precursor processing. Genes Dev..

[31] Schwartz Y.B., Kahn T.G., Nix D.A., Li X.-Y., Bourgon R., Biggin M., Pirrotta V. (2006). Genome-wide analysis of Polycomb targets in Drosophila melanogaster. Nat. Genet..

[32] Noordermeer D., Leleu M., Splinter E., Rougemont J., De Laat W., Duboule D. (2011). The Dynamic Architecture of *Hox* Gene Clusters. Science.

[33] Frapporti A., Pina C.M., Arnaiz O., Holoch D., Kawaguchi T., Humbert A., Eleftheriou E., Lombard B., Loew D., Sperling L. (2019). The Polycomb protein Ezl1 mediates H3K9 and H3K27 methylation to repress transposable elements in Paramecium. Nat. Commun..

[34] Miró-Pina C., Charmant O., Kawaguchi T., Holoch D., Michaud A., Cohen I., Humbert A., Jaszczyszyn Y., Chevreux G., Del Maestro L. (2022). Paramecium Polycomb repressive complex 2 physically interacts with the small RNA-binding PIWI protein to repress transposable elements. Dev. Cell.

[35] Montgomery S.A., Tanizawa Y., Galik B., Wang N., Ito T., Mochizuki T., Akimcheva S., Bowman J.L., Cognat V., Maréchal-Drouard L. (2020). Chromatin Organization in Early Land Plants Reveals an Ancestral Association between H3K27me3, Transposons, and Constitutive Heterochromatin. Curr. Biol..

[36] Zhao X., Xiong J., Mao F., Sheng Y., Chen X., Feng L., Dui W., Yang W., Kapusta A., Feschotte C. (2019). RNAi-dependent *Polycomb* repression controls transposable elements in *Tetrahymena*. Genes Dev..

[37] Creyghton M.P., Cheng A.W., Welstead G.G., Kooistra T., Carey B.W., Steine E.J., Hanna J., Lodato M.A., Frampton G.M., Sharp P.A. (2010). Histone H3K27ac separates active from poised enhancers and predicts developmental state. Proc. Natl. Acad. Sci. USA.

[38] Lavarone E., Barbieri C.M., Pasini D. (2019). Dissecting the role of H3K27 acetylation and methylation in PRC2 mediated control of cellular identity. Nat. Commun..

[39] Karmodiya K., Krebs A.R., Oulad-Abdelghani M., Kimura H., Tora L. (2012). H3K9 and H3K14 acetylation co-occur at many gene regulatory elements, while H3K14ac marks a subset of inactive inducible promoters in mouse embryonic stem cells. BMC Genom..

[40] Boyd J.B., Sakaguchi K., Harris P.V. (1990). mus308 mutants of Drosophila exhibit hypersensitivity to DNA cross-linking agents and are defective in a deoxyribonuclease. Genetics.

[41] Leonhardt E.A., Henderson D.S., Rinehart J.E., Boyd J.B. (1993). Characterization of the mus308 gene in Drosophila melanogaster. Genetics.

[42] Seki M., Marini F., Wood R.D. (2003). POLQ (Pol), a DNA polymerase and DNA-dependent ATPase in human cells. Nucleic Acids Res..

[43] Park J.W., Parisky K., Celotto A.M., Reenan R.A., Graveley B.R. (2004). Identification of alternative splicing regulators by RNA interference in *Drosophila*. Proc. Natl. Acad. Sci. USA.

[44] Herold N., Will C.L., Wolf E., Kastner B., Urlaub H., Lührmann R. (2009). Conservation of the Protein Composition and Electron Microscopy Structure of *Drosophila melanogaster* and Human Spliceosomal Complexes. Mol. Cell. Biol..

[45] Akhtar A., Becker P.B. (2000). Activation of Transcription through Histone H4 Acetylation by MOF, an Acetyltransferase Essential for Dosage Compensation in Drosophila. Mol. Cell.

[46] Tie F., Banerjee R., Stratton C.A., Prasad-Sinha J., Stepanik V., Zlobin A., Diaz M.O., Scacheri P.C., Harte P.J. (2009). CBP-mediated acetylation of histone H3 lysine 27 antagonizes *Drosophila* Polycomb silencing. Development.

[47] Tie F., Banerjee R., Conrad P.A., Scacheri P.C., Harte P.J. (2012). Histone Demethylase UTX and Chromatin Remodeler BRM Bind Directly to CBP and Modulate Acetylation of Histone H3 Lysine 27. Mol. Cell. Biol..

[48] Birve A., Sengupta A.K., Beuchle D., Larsson J., Kennison J.A., Rasmuson-Lestander A., Müller J. (2001). *Su(z)12*, a novel *Drosophila* Polycomb group gene that is conserved in vertebrates and plants. Development.

[49] Margueron R., Reinberg D. (2011). The Polycomb complex PRC2 and its mark in life. Nature.

[50] McAvera R.M., Crawford L.J. (2020). TIF1 Proteins in Genome Stability and Cancer. Cancers.

[51] Filion G.J., van Bemmel J.G., Braunschweig U., Talhout W., Kind J., Ward L.D., Brugman W., de Castro I.J., Kerkhoven R.M., Bussemaker H.J. (2010). Systematic Protein Location Mapping Reveals Five Principal Chromatin Types in Drosophila Cells. Cell.

[52] Ho J.W.K., Jung Y.L., Liu T., Alver B.H., Lee S., Ikegami K., Sohn K.-A., Minoda A., Tolstorukov M.Y., Appert A. (2014). Comparative analysis of metazoan chromatin organization. Nature.

[53] Bilodeau S., Kagey M.H., Frampton G.M., Rahl P.B., Young R.A. (2009). SetDB1 contributes to repression of genes encoding developmental regulators and maintenance of ES cell state. Genes Dev..

[54] Voigt P., LeRoy G., Drury W.J., Zee B.M., Son J., Beck D.B., Young N.L., Garcia B.A., Reinberg D. (2012). Asymmetrically Modified Nucleosomes. Cell.

[55] Ma X., Yang T., Luo Y., Wu L., Jiang Y., Song Z., Pan T., Liu B., Liu G., Liu J. (2019). TRIM28 promotes HIV-1 latency by SUMOylating CDK9 and inhibiting P-TEFb. eLife.

[56] Li J., Xi Y., Li W., McCarthy R.L., Stratton S.A., Zou W., Dent S.Y., Jain A.K., Barton M.C. (2017). TRIM28 interacts with EZH2 and SWI/SNF to activate genes that promote mammosphere formation. Oncogene.

[57] Cheng B., Ren X., Kerppola T.K. (2014). KAP1 Represses Differentiation-Inducible Genes in Embryonic Stem Cells through Cooperative Binding with PRC1 and Derepresses Pluripotency-Associated Genes. Mol. Cell. Biol..

[58] Carré C., Szymczak D., Pidoux J., Antoniewski C. (2005). The Histone H3 Acetylase dGcn5 Is a Key Player in *Drosophila melanogaster* Metamorphosis. Mol. Cell. Biol..

[59] Agricola E., Randall R.A., Gaarenstroom T., Dupont S., Hill C.S. (2011). Recruitment of TIF1γ to Chromatin via Its PHD Finger-Bromodomain Activates Its Ubiquitin Ligase and Transcriptional Repressor Activities. Mol. Cell.

[60] Tsai W.-W., Wang Z., Yiu T.T., Akdemir K.C., Xia W., Winter S., Tsai C.-Y., Shi X., Schwarzer D., Plunkett W. (2010). TRIM24 links a non-canonical histone signature to breast cancer. Nature.

[61] Ziv Y., Bielopolski D., Galanty Y., Lukas C., Taya Y., Schultz D.C., Lukas J., Bekker-Jensen S., Bartek J., Shiloh Y. (2006). Chromatin relaxation in response to DNA double-strand breaks is modulated by a novel ATM- and KAP-1 dependent pathway. Nat. Cell Biol..

[62] White D.E., Negorev D., Peng H., Ivanov A.V., Maul G.G., Rauscher F.J. (2006). KAP1, a Novel Substrate for PIKK Family Members, Colocalizes with Numerous Damage Response Factors at DNA Lesions. Cancer Res..

[63] Goodarzi A.A., Kurka T., Jeggo P.A. (2011). KAP-1 phosphorylation regulates CHD3 nucleosome remodeling during the DNA double-strand break response. Nat. Struct. Mol. Biol..

