# Supplementary figures and images for: Impact of Germline Depletion of Bonus on Chromatin State in Drosophila Ovaries
Source: Cells. 2023 Nov 15;12(22):2629. doi: 10.3390/cells12222629 (PMC10670193; doi:10.3390/cells12222629)

Figure S1

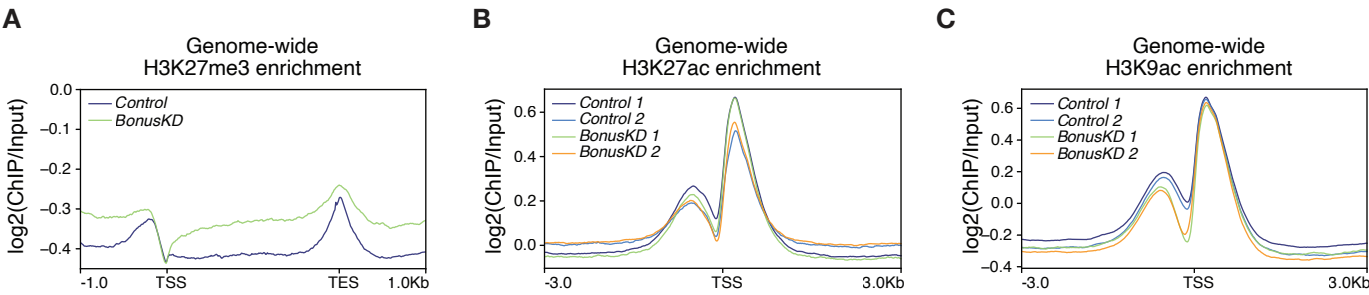

Supplement: Supplementary file 1 [file cells-12-02629-s001.zip › cells-2697608-supplementary.pdf]
